# Using Intervention Mapping to develop the Parents as Agents of Change (PAC^©^) intervention for managing pediatric obesity

**DOI:** 10.1186/s13104-016-2361-3

**Published:** 2017-01-13

**Authors:** Geoff D. C. Ball, Aislin R. Mushquash, Rachel A. Keaschuk, Kathryn A. Ambler, Amanda S. Newton

**Affiliations:** 1 Department of Pediatrics, University of Alberta, Room 4-515, Edmonton Clinic Health Academy, 11405-87th Ave, Edmonton, AB T6G 1C9 Canada; 2 Department of Pediatrics, University of Alberta, Edmonton, AB Canada; 3 Department of Psychology, Lakehead University, 955 Oliver Rd, Thunder Bay, ON P7B 5E1 Canada; 4 #780 Princeton Place, 10339-124th St, Edmonton, AB T5N 3W1 Canada; 5 Health Technology & Service Policy, Research & Innovation Branch, Strategic Planning & Policy Development Division, Alberta Health, 18th Floor, ATB Building, 10025 Jasper Ave, Edmonton, AB T5J 1S6 Canada; 6 Department of Pediatrics, University of Alberta, Room 3-526, Edmonton Clinic Health Academy, 11405-87th Ave, Edmonton, AB T6G 1C9 Canada

**Keywords:** Child, Cognitive therapy, Obesity, Parents, Pediatric obesity, Weight reduction programs

## Abstract

**Background:**

Pediatric obesity has become increasingly prevalent over recent decades. In view of the psychosocial and physical health risks, and the high likelihood that children with obesity will grow to become adults with obesity, there is a clear need to develop evidence-based interventions that can be delivered in the health care system to optimize the health and well-being of children with obesity and their families. The aim of this paper is to describe the development, implementation, and planned evaluation of a parent-based weight management intervention designed for parents of 8–12 year olds with obesity.

**Methods/results:**

The principles of Intervention Mapping (IM) were used to develop an intervention called *Parents as Agents of Change* (PAC^©^). From 2006 to 2009, an environmental scan plus qualitative (individual interviews with parents and children), quantitative (medical record reviews), and literature review data were collected to gain broad insight into family factors related to pediatric obesity and its management. Theoretical frameworks and empirical evidence guided curriculum development, which was founded primarily on the tenets of family systems theory and cognitive behavioral theory. PAC was developed as a manualized, 16-session, group-based, health care professional-led intervention for parents to address individual, family, and environmental factors related to the management of pediatric obesity. The intervention was refined based on feedback from local and international experts, and has been implemented successfully in a multi-disciplinary weight management centre in a children’s hospital.

**Conclusion:**

IM provided a practical framework to guide the systematic development of a pediatric weight management intervention for parents of children with obesity. This logical, step-by-step process blends theory and practice and is broadly applicable in the context of obesity management intervention development and evaluation. Following intervention development, the PAC intervention was evaluated within a randomized clinical trial.

*Trial registration* NCT01267097; clinicaltrials.gov

## Background

Pediatric obesity is a major public health concern internationally [1]. Along with impacting 10–20% of children in the United States [2] and Canada [3], excess weight is associated with various medical (e.g., insulin resistance [4]), psychological (e.g., poor self-esteem [5]), and relational problems (e.g., bullying [6, 7]) and tends to persist into adulthood along with its associated comorbidities [8]. With these issues in mind, it is imperative to develop, implement, and evaluate weight management interventions designed to optimize the health of children with obesity and their families.

Comprehensive, family-based interventions that target lifestyle behaviors are effective in improving children’s weight status and weight-related health risks [9–11]. However, the extent to which parents and their children actively participate in family-based interventions has varied within and between intervention studies to date. A growing body of evidence has demonstrated that interventions designed for parents exclusively, often referred to as Parents as Agents of Change (PAC^©^) programs, are both efficacious and effective [12–17]. There are important practical advantages to parent-only interventions over parent-and-child or one-on-one care, which include reduced staffing, materials, physical space, money, and (potentially) less stigmatization for the child [18–20]. In addition, parents play leadership roles in families, so interventions designed for parents to help them establish and maintain healthy home environments and serve as positive role models for their children is a logical and appropriate intervention approach.

Despite evaluation of and support for PAC interventions, scant details are available in the literature regarding intervention rationale, theoretical underpinnings, curricular components, decision-making processes, and evaluation plans. This is a limitation since decision-makers (e.g., physicians, managers, administrators) within health care systems strive to provide evidence-based, effective and efficient health services to improve the health of children with obesity and their families [21]. The need for such interventions is reflected, at least within Canada, in the number of family-based, clinical pediatric weight management centres that have emerged in recent years [22]. In the absence of details regarding how interventions were conceived, developed, and evaluated, stakeholders are left with inadequate information to inform future planning; in turn, this can have a negative impact on program initiation and sustainability. This concern is particularly relevant for chronic diseases such as obesity since successful management is a challenge for families and clinicians, requiring long-term planning, support, and monitoring. The purpose of this manuscript is to describe the process we used to develop a PAC lifestyle intervention for parents of 8–12 year olds with obesity that could be offered within the health care system.

## Methods and results

**Fig. 1:**
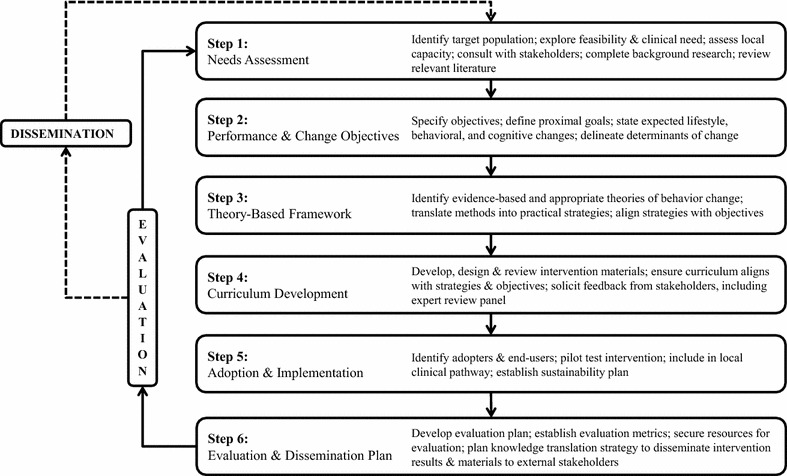
Adapted Intervention Mapping process used to develop the Parents as Agents of Change^©^ weight management intervention for parents of children with obesity

The PAC intervention was developed and implemented using strategies consistent with Intervention Mapping (IM) [23] (Fig. 1). This process ensures that newly developed interventions are grounded in theory and empirical data; evidence suggests this approach to intervention development is associated with improved effectiveness [24]. IM involves a series of sequential steps beginning with a needs assessment (Step 1) and creating performance objectives, determinants, and change objectives by specifying who and what will change as a result of the intervention (Step 2). Then, theory-based methodologies and strategies are selected (Step 3), the intervention is designed and organized (Step 4), and the intervention is adopted and implemented (Step 5). The last step involves finalizing an evaluation plan (Step 6). For the sake of parsimony, and to remain consistent with previous reports describing the process of intervention development [25, 26], our methodological approach and associated outcomes are combined and described in detail below.

### Step 1—needs assessment

We conducted a needs assessment with various stakeholders (e.g., children, parents, providers, researchers, policy makers) to explore issues related to offering pediatric weight management care. In 2006, while our clinical program was under development, administrators at the local children’s hospital commissioned a province-wide environmental scan of health services and resources for children with obesity and their families. This process involved interviews with more than 40 stakeholders (e.g., health care providers, researchers) and findings revealed there were no dedicated clinical health services for pediatric obesity in the province of Alberta (population: ~4,000,000) and therefore no local infrastructure to build upon. Many interviewees underscored the importance of providing pediatric weight management care for children and hoped that our new centre could provide these services and provide guidance for health care professionals working in primary care and community-based settings.

In 2006 and 2007, we collected qualitative data regarding treatment preferences from parents (n = 21) and children (n = 20) who were waiting to receive care from our program [27]. Parents and children were interviewed individually (and separately). We collected information across a broad range of areas including nutrition, peers, parenting strategies, physical activity, screen time, school, treatment preferences, and past experiences receiving weight management care. At the family level, parents expressed difficulties with maintaining consistency in their efforts to support change in their children’s lifestyle behaviors and tended to vacillate in their parenting strategies between extremes (i.e., leniency and control). Although parents often express positive intentions, such inconsistent parenting behaviors are associated with pediatric obesity and are increasingly targeted in obesity prevention and management interventions [28, 29]. At the health care system level, families expressed a desire for family-oriented care and support from health care professionals to help them achieve and maintain healthy family changes. Overall, these qualitative data highlighted the complexity of pediatric obesity and offered insight into proximal familial and distal system-level factors, which may influence the likelihood that children and their families will be successful in their weight-management efforts, and that we needed to consider in our intervention development.

In 2007, we studied the lifestyle behaviors of children referred to our weight management centre [30]. Data were collected before children and their families received weight management services and revealed that most children did not achieve lifestyle recommendations prior to receiving care. For instance, the minority (i) consumed the recommended servings/day of vegetables and fruit (14.1%), (ii) participated in the recommended amount of physical activity time (7.4%) or accumulated adequate steps/day (4.1%), (iii) adhered to daily screen time guidelines (22.7%), and (iv) met nightly sleep time goals (47.4%). We recently published the anthropometric and lifestyle characteristics of parents of children with obesity before families initiated weight management care [31]. Most parents satisfied criteria for overweight (BMI ≥ 25 kg/m^2^; n = 61; ~25%) or obesity (BMI ≥ 30 kg/m^2^; n = 139; ~57%), few (<20%) consumed the recommended servings/day of vegetables and fruit, many (>60%) consumed excessive sugar-sweetened beverages, and the minority (~20%) achieved the recommended physical activity recommendations. Results among parents were consistent with those among children and suggested that families were not achieving lifestyle behavior recommendations prior to receiving health services. Therefore, our data suggested the need for a family-oriented intervention designed to help parents and their children with obesity to improve lifestyle behaviors (e.g., increase dietary quality, increase physical activity, and reduce physical inactivity), which are linked to improvements in weight management and reductions in health risk [9, 32].

Finally, we completed a literature review and synthesis of published research on the management of pediatric obesity and, in particular, on the efficacy and effectiveness of parent-based interventions in managing obesity in children. Since the mid-2000s, numerous clinical practice guidelines [33, 34], expert recommendations [35, 36], systematic reviews [10, 11], and position statements [37, 38] were published regarding pediatric weight management. Universally, all of these reports underscore the fundamental role that parents play in facilitating children’s successful weight management. At the time we began developing the PAC intervention, research focusing on parents exclusively to help their children and families make healthy lifestyle and behavioral changes was unique in the field. This work was led by Moira Golan and colleagues [16, 17, 19, 20] and influenced our intervention development to a great extent. Since then, several additional reports [13–15] have supported parent-only approaches to managing pediatric obesity.

### Step 2—performance and change objectives

The overall goal of Step 2 is to define specific intervention goals that are developed from specific performance objectives and determinants of behavior change [39]. We used the results of Step 1 to inform our performance and change objectives. Performance objectives are the effects of the intervention on the target group with respect to what should be learned or specific behavior(s) that should be modified. Matrices that combine these performance objectives with specific determinants of pediatric obesity were developed to define intervention goals, namely specific learning objectives (i.e., what the target group needs to learn or acquire) and specific change objectives (i.e., what the target group needs to change).

As an overarching aim, our intervention goal was to improve the health and well-being of children with obesity by helping parents to make healthy changes within their families. More specifically, and consistent with the mandate of our local pediatric weight management centre, the primary goal of our intervention was to improve the weight status of children with obesity by working with their parents exclusively to make cognitive, behavioural, and lifestyle changes that enable weight management success. Although accomplishing a goal of substantial weight loss could reduce many of the physical and psychosocial health risks that accompany obesity in childhood, few children are able to achieve this ideal. Operationally, successful pediatric weight management can represent either a stabilization or a reduction in BMI z-scores over time [35]. Even modest improvements in children’s weight status are associated with positive changes in cardiometabolic risk factors [40–42] suggesting that dramatic weight reductions are not required to improve health outcomes.

We observed that lifestyle behaviors related to obesity were common in both parents and their children, and that an intervention designed to improve cognitive, behavioral, and lifestyle habits in families was meritorious. Subsequently, we organized performance objectives (Table 1) to guide how parents would achieve this aim within the intervention. In generating the performance objectives, we met with an inter-professional team of health care professionals who offer pediatric weight management care to children and families at our local children’s hospital. Their clinical experience and training in nutrition, health promotion, physical activity, exercise physiology, mental health, nursing, endocrinology, and pediatric medicine complemented our research expertise. We shared the results of our needs assessment, local research studies, and literature review with this group to inform our ongoing discussions. After considering performance objectives and the determinants of pediatric obesity, the group examined which lifestyle habits were most amenable to change and then generated a list to consider for inclusion in the intervention. Given the fundamental roles of diet and physical activity in determining energy balance and weight management success, we established six lifestyle-related change objectives (three diet- and three physical activity-related; see Table 2). We chose these goals because: (i) of their impact on obesity and weight management, (ii) they offered families tangible targets they could work on together, and (iii) families were unlikely to be achieving all of these targets at presentation, so all families would be able to identify at least one lifestyle behavior that they could address [30, 31].

**Table 1:** Performance objectives for the Parents as Agents of Change (PAC^©^) intervention

Performance objectives were designed for parents to help them make changes (lifestyle, behavioral, cognitive, interpersonal) to enable weight management for their children
Parents learn the causes and consequences of childhood obesity
Parents learn how they can facilitate and reinforce changes
Parents learn about how they can serve as positive role models and leaders within their family
Parents can identify and discuss real and perceived barriers to make positive changes
Parents can discuss and prioritize solutions by problem-solving plans to make positive changes
Parents can discuss and implement strategies to make and maintain positive changes
Parents can explore how their thoughts and feelings influence their behaviors in helping their children to make positive changes

**Table 2:** Lifestyle goals within the Parents as Agents of Change (PAC^©^) intervention

Diet	Daily vegetable and fruit intake: ≥5 servings [74, 75]
	Daily grain product intake: ≥50% of servings as whole grains [74]
	Daily sugar-sweetened beverage intake: 0 servings [35]
Physical activity	Daily steps: ≥12,000 (girls); ≥15,000 (boys) [76]
	Daily moderate-to-vigorous physical activity: ≥90 min [77]
Sedentary activity	Daily leisure time screen time: ≤90 min [77]

The lifestyle goals of PAC^©^ were based on our team’s interpretation of the best available evidence for pediatric weight management at the time the intervention was developed

### Step 3—theory-based framework

The goal of Step 3 is to identify and select theory-based models that are relevant to families, obesity, and behavior change, and then translate them into practical intervention tools used to target the identified change objectives. Given the role of the family in children’s health behaviors, developing theoretically-driven interventions that incorporate parents as leaders within their families is vitally important [43]. With this in mind, elements were taken from well-established theoretical models to develop the PAC intervention; specifically, we drew upon family systems theory (FST) [44, 45] and cognitive behavior therapy (CBT) [46, 47]. Relevant details from each theory are described below.

### Family systems theory

According to FST, parenting style is often conceptualized as the emotional climate in which parenting practices occur [48] and parenting style can influence children’s lifestyle behaviors and weight as it represents the context in which specific parenting practices and lifestyle behaviors are presented [49]. Three distinct parenting styles are discussed in the literature, namely: authoritative, authoritarian, and permissive [50]. Authoritative parenting incorporates shared decision-making, high expectations paired with warmth and compassion, and setting and enforcing appropriate limits and consequences. Authoritative parenting is associated with better outcomes including more healthful eating practices and lower rates of obesity in children [51, 52].

Similar to authoritative parenting, authoritarian parenting is characterised by high expectations; however, involves little warmth and compassion and instead involves high levels of discipline and rigid control. Authoritarian parenting is associated with more adverse outcomes as children are overly controlled by their parents and not able to develop autonomy and appropriate self-regulation skills [53].

Permissive (or indulgent) parenting is typified by little to no demands placed on the child paired with high levels of warmth and compassion. Permissive parenting styles can have a detrimental effect on a family’s ability to make changes in their lifestyle behaviors and can promote unhealthy lifestyle patterns [54]. For example, parents using a permissive parenting style often do not exert expectations on their children and may not provide the guidance that is needed for children to develop self-regulation in their eating and their activity behaviors [55].

Along with characteristic parenting styles, family systems theory suggests that dysfunction in the familial organization or structure contributes to dysfunction in the child, and that children’s behaviors should be conceptualized in terms of the entire family system [56]. In his work, Minuchin suggests children who experience psychosomatic symptoms or disordered eating behaviors often have families that are enmeshed, conflict avoidant, and lacking in conflict resolution. In such families, the illness and symptom is often maintained as a means of preserving homeostasis or status quo in the family system [57, 58].

Many tenets from family system theory can be applied to understanding pediatric obesity. Our intervention focuses on helping parents to work towards developing more helpful parenting approaches (e.g., authoritative parenting) and recognizing behaviors within their family structure that (inadvertently or unintentionally) maintain unhealthy habits. For example, one PAC session focuses on positive parenting partnerships and practical skills such as setting limits with respect lifestyle behaviors, maintaining appropriate boundaries, and improving communication among family members. In addition to the specific content on parenting practices, parents are encouraged throughout the intervention to make use of these new parenting techniques as they implement other changes in the family.

### Cognitive behavioral therapy

The collective body of evidence derived from clinical research supports the application of cognitive behavioral therapy (CBT)-based interventions in weight management for both adults [59–61] and children [62, 63]. CBT is a theoretically-based treatment approach that highlights the relationship between cognitions (thoughts), feelings, and behaviors, and utilizes techniques involving motivational enhancement, goal-setting, problem-solving, and knowledge/skill acquisition that can facilitate sustainable behavior changes [46, 47]. In our intervention, skills gained through CBT are designed to help parents identify and change the parenting mechanisms that maintain their children’s current lifestyle habits. Our CBT-trained health care professionals work with parents to link knowledge, attitudes, thoughts, and feelings to behaviors and facilitate setting incremental goals that build week-to-week and are individualized to match the degree of parental motivation to change.

Consistent with a CBT framework, parents in our intervention are encouraged to consider their attitudes towards food, physical activity, and physical inactivity. Food can be modeled dichotomously as *good* or *bad*, used as a means of self-soothing or reward, or withheld as a punishment. Similarly, families can view engaging in or avoiding physical activity as *good* or *bad*, as a competitive activity, as a punishment, or as a means to reduce stress and anxiety or increase skill and competence. For these reasons, we included training for parents on examining their beliefs about food and activity behaviors and how these behaviors are encouraged in the home. Raising parental awareness of their own beliefs about food and activity behaviors can help parents break the transmission of these beliefs to their children.

CBT encourages consistent participation and collaboration between health care professionals and participants. We used a Socratic questioning approach to help parents find their own answers to problems which is linked more closely with intrinsic motivation and can lead to sustainable behavior change [64], instead of having questions answered or problem-solved by a health care professional.

As mentioned above, PAC is deliberate in promoting parental adoption of more authoritative parenting strategies and recognizing patterns in the home. CBT techniques can also be helpful in improving such parenting strategies and improving parent–child interactions [65, 66]. During PAC, health care professionals work with parents who have more permissive (e.g., makes few demands) or authoritarian (e.g., restricts child autonomy) parenting styles to develop authoritative parenting skills.

### Step 4—curriculum development

The aim of Step 4 is to describe the scope and components of the intervention. To achieve this aim, we completed a series of activities to develop the PAC curriculum. First, we held a 3-day, facilitated workshop with a team of clinicians (e.g., pediatrician, nurse practitioner, dietitian, exercise specialist, health promoter) that offers health services for obesity management at our local children’s hospital. The purpose of these sessions was to solicit input from front-line clinicians to determine the topics and issues for inclusion in the curriculum, perspectives that were complemented by information retrieved from the published literature as well as online descriptions of existing weight management services. It is important to mention that PAC curriculum developers were also a part of the weight management team, serving as clinical psychologist (RAK) and program director (GDCB). Second, based on the information gathered and consistent with the objectives and theoretical underpinnings of the intervention, we created 16 inter-related and manualized sessions (each 60–90 min in duration) that could be delivered in a group setting by health professionals to parents of children with obesity. The group-based format met our need to offer health services for families in a way that optimized our available physical space, personnel, and funding. Topics covered in the PAC curriculum were relevant to weight management including nutrition, physical activity, sedentary behavior, parenting, communication, mental health, and behavior change. It is worth noting that a wide range of topics was discussed during the workshop; however, we gained consensus from the group on the issues thought to be most important for parents to make lifestyle, behavioral, and cognitive changes to enable weight management in children. Third, complementing the information included in each individual session, we embedded practical activities that were consistent with CBT. For instance, at the start of each session, health care providers delivering PAC would initiate a facilitated discussion with parents about their goals for the past week and encourage them to share their successes and challenges with the group. This activity reinforces aspects of CBT (linking thoughts and feelings with behaviors) and provides an opportunity for parents to learn from one another as well as validate experiences among parents since many shared common situations and challenges in making and maintaining positive changes in their families. Additional materials were developed that aligned with intervention content, including a (i) *PAC Snapshot* template that can be populated with clinical (e.g., systolic blood pressure), anthropometric (e.g., BMI percentile), and lifestyle (e.g., steps/day) data from children pre- and post-intervention; this resource highlights potential areas of interest for families to address during the intervention as well as highlights changes in modifiable outcomes (e.g., nutrition, physical activity) from the start to the end of the intervention; (ii) *PAC Goal*-*Setting* template that adheres to well-established principles (specific, measurable, attainable, relevant, and timely) [67] and is used by parents on a week-to-week basis to structure their goal-setting plans; and (iii) *PAC Tracking Sheet* template that can be used by both parents and children to monitor lifestyle habits, including recording steps/day or intake of vegetables and fruit.

Intervention materials were developed using Microsoft PowerPoint^©^ and Word^©^. PowerPoint^©^ was used to create two complementary slide decks—one for health care professionals (PAC leaders) and one for parents, which are printed and organized in binders. Both slide decks include the same presentation slides that are shown to the group, but the leaders’ version also includes details (viewable in the notes section) in each individual slide on the session purpose, resources needed to deliver the session, information to emphasize for parents during the presentation, and literature cited/evidence in support of the content. The parents’ version also includes space for note-taking and for completing specific activities that are aligned with the session content.

The 16 sessions in the PAC interventions were organized purposefully. In our experience, when families decide to seek health services for pediatric weight management, they often present with questions related to medical concerns, nutrition, and physical activity. This observation led us to provide sessions on obesity (e.g., definitions, causes, consequences), nutrition (e.g., eating meals away from home, portion sizes), and physical activity (e.g., activity versus exercise, screen time) in the first half of the intervention, which helped clinicians to engage with parents given that these topics were often expected by families and to allow families to work on lifestyle and behavioral changes over time. Sessions that focus on other topics (e.g., parenting, communication, time management, self-esteem) were delivered in the second half since these topics can be more secondary for parents, but allow leaders and parents to relate earlier content-related issues (e.g., eating more vegetables and fruit) to later process-related topics (e.g., improving time management skills to enable healthier meals).^1^ Further, we incorporated a number of different activities across the 16 sessions to capitalize on different adult learning styles and preferences, which included group discussions, brain-storming, interactive and experiential learning, paired conversations, and individual private activities. In general, longer interventions result in a greater likelihood of success in weight management [10]; however, intervention length needs to correspond with most families’ readiness, willingness, and ability so that enrolment and participation are optimized, which includes considering work, school, and seasonal or intermittent commitments such as holidays and exams. We believe there is nothing extraordinary about 16 sessions (in lieu of, for instance, 15 or 17 sessions) that can be delivered over 16 consecutive weeks. This duration was informed by our local weight management clinic, which could offer PAC to families twice annually (September to December; January to April), and was long enough for parents to progress through a series of stages believed to be important for small groups (forming, storming, norming, performing, and adjourning) [68]. We also relied on evidence suggesting that this intervention duration was likely long enough for children with obesity to experience improvements in weight and health [69, 70].

Once the PAC intervention was drafted, we commissioned an international expert review panel with members from Canada, the United States, and Israel to review all PAC materials. Individuals possessed a blend of clinical and research expertise in CBT, obesity, nutrition, as well as intervention development and evaluation. Upon the review of the intervention, panel members provided us with a critical evaluation of our intervention, which led to subsequent refinements in concepts, content, and clarity to PAC materials.

### Step 5—adoption and implementation

For Step 5, we worked with the team of health care professionals offering weight management services to families at our local children’s hospital to adopt the PAC intervention into their clinical pathway. Because this team helped us to develop the content of the PAC intervention, this step was a logical extension and reflected their ongoing participation. At the time we started to develop the PAC intervention in 2006, the only health services available for weight management at our children’s hospital included individual care; therefore, the group-based PAC intervention offered the clinic and families a new treatment modality that complemented existing services. Despite a lack of data on the efficacy and effectiveness of the PAC intervention, the team agreed to offer it as a clinical service and help evaluate it for feasibility, acceptability, and satisfaction.

From 2007 to 2009, we pilot-tested the PAC intervention with 58 families, which included a total of 72 parents who were recruited through our local pediatric weight management clinic (unpublished data). In partnership with clinical and administrative members of the clinic, we recruited and enrolled parents of children (8–12 years old) with obesity who were participating in the clinic. Families were enrolled in six individual cohorts that varied in size from eight to 14 parents. The attrition rate was 25% and, on average, parents attended 13 out of 16 sessions. The intervention was delivered by 14 different intervention leaders (two leaders per cohort), which included health care professionals and clinical trainees with expertise in exercise physiology, health promotion, nursing, nutrition, psychology, and social work. Each session took between 60 and 90 min to deliver. Within this pilot, we offered two versions of the PAC intervention; the CBT version of PAC was delivered by individuals with experience and training in CBT whereas a companion version of the PAC intervention, which included the same goals, duration, and content, was offered in a more traditional, didactic format that did not include CBT-specific activities (e.g., linking thoughts and feelings to behaviors). The non-CBT version of PAC was offered by health care professionals and clinical trainees who did not possess training in CBT. We piloted two versions of PAC since our goal (beyond the pilot phase) was to secure external research funding to compare the effectiveness of two parent-based interventions for managing pediatric obesity.

Before the start of each session, our research leadership team (GDCB, ASN, RAK) met with the PAC leaders for ~30 min to discuss intervention delivery and family process issues. These regular sessions not only provided us with direct feedback from PAC deliverers, which informed intervention modifications that were made while the PAC intervention was being delivered week-to-week, but gave us the opportunity to mentor and support our clinical team members. Each session was also followed by a short debriefing period that included cataloguing issues that arose during the session and required follow-up prior to the next session (e.g., questions from parents that extended beyond intervention content and leader knowledge; unexpected parent group member absences). On a week-to-week basis, we also incorporated modest contingency rewards (e.g., free family admission passes to local recreation center) to capitalize on families’ extrinsic motivation for changing lifestyle habits, which families were eligible for when they returned their completed *PAC Tracking Sheets*. Other elements were incorporated into the PAC intervention that were designed to enhance families’ participation and perceived value in the program. For instance, (i) all families received two pedometers that they could use to track their daily steps; (ii) PAC leaders and parents were encouraged to share educational resources with their group by placing supplementary materials (e.g., recipes, health tips, books and brochures) on a community resource table, which encouraged participants to be active in sharing information and experiences; and (iii) families were encouraged to contact PAC leaders or the clinic secretary if they believed they would benefit from additional support through one-on-one consultations with clinicians (e.g., dietitian, exercise specialist, psychologist, pediatrician) from the weight management clinic.

We did not establish specific metrics regarding feasibility, but our collective experience revealed that the PAC intervention was easy to add to the clinical pathway of health services for weight management and the health care professionals valued the intervention as a treatment option for families, which complemented the one-on-one care they already offered. To gain perspectives from parents who completed the PAC intervention, we had them complete structured exit surveys at the completion of the curriculum to provide us with feedback on a variety of intervention elements (e.g., content, duration, timing, likes/dislikes, and recommendations for changes), which offered insights related to acceptability and satisfaction. Parents reported that the material was helpful in addressing their children’s weight management and found the content of PAC was useful for them and their families. Based on their feedback, minor refinements were made to the content and organization of the final version of the PAC intervention. Overall, parents’ responses (n = 72) were favorable with most reporting they believed the 60–90 min session length (54/72; 75%) and 16-session duration (62/72; 86%) were *just right*. Further, most parents *agreed* or *strongly agreed* that they (i) were satisfied with the PAC intervention (68/72; 94%), (ii) had their expectations met (61/72; 85%), and (iii) were able to make healthy lifestyle changes as a result of the program (70/72; 97%).

### Step 6—evaluation and dissemination

In step six, we built on the experience we gained during the pilot-testing phase of intervention development and refinement. We secured external research funding to evaluate the PAC intervention within a two-armed, parallel, single-blinded, superiority, randomized clinical trial [71]. We chose a study design that allowed us to compare the effectiveness of the PAC intervention based on principles of CBT *versus* a non-CBT version of PAC in targeting the diet, physical activity, and sedentary activity change objectives mentioned in step 2 (see Table 2). The non-CBT version of PAC was similar in content and structure, but lacked components of CBT that link thoughts and feelings to behaviors. The outcomes assessed at pre-intervention, post-intervention, 6-, and 12-months follow-up will include the child’s BMI z-score, and cardiometabolic, lifestyle, and psychosocial variables in children and parents. Our study retained methodological rigour while adapting to the local needs of the pediatric weight management clinic to offer timely health services to families. We will finalize and submit the results of our trial for publication in the coming months, data that follow a complementary study that explored families’ experiences and perceptions related to the PAC intervention [72].

From the onset of this research, we believed that other clinicians and health care administrators may be interested in offering the PAC intervention to families in other regions. Through word of mouth, this has occurred, with colleagues from three other communities/clinics slated to provide the PAC program over the coming year. We shared information about PAC within our local children’s hospital administration via briefing notes and provincially through Alberta Health Services’ website and a provincial network of clinicians and administrators whose work focuses on preventing and managing pediatric obesity. Although we did not establish a formal dissemination plan, we partnered recently with the Canadian Obesity Network (www.obesitynetwork.ca) to use their existing infrastructure and network of members to share information about the PAC intervention to a broader audience of stakeholders.

## Discussion and conclusions

Many children are living with obesity and associated comorbidities. Parents play a key role in leading family lifestyle and behavioral changes; therefore, PAC interventions are promising strategies for helping to manage pediatric obesity. Despite empirical support for PAC interventions, little information is available in published literature and elsewhere regarding the rationale and development of these programs. The purpose of this manuscript was to describe the development, implementation, and planned evaluation of a new PAC weight management intervention using an IM framework. Our PAC intervention addresses individual, family, and environmental factors related to managing pediatric obesity by involving parents of 8–12 year olds with obesity in a 16-session, group-based, clinician-led program. Information relevant to weight management including nutrition, physical activity, sedentary behavior, parenting, communication, mental health, and behavior change is provided throughout the sessions. Parents are supported throughout the program in setting realistic goals, self-monitoring, and problem solving.

The IM approach used in developing the PAC intervention has many strengths. Specifically, this approach allowed the research team to systematically incorporate information from stakeholders (including parents, children, providers, researchers, and policy makers), theoretical models of behavior change and obesity management, and existing empirical data. Not only is IM associated with improved effectiveness [24], but it also promotes capacity building as other healthcare teams and researchers can benefit from the detailed account of the intervention development. Despite the strengths of IM, this approach is resource intensive and time consuming [26, 73], which may be a barrier to smaller healthcare teams. Moreover, the PAC program discussed here was developed within a specific clinic, so it is possible that data obtained (e.g., needs expressed from stakeholders) might differ in other jurisdictions. All PAC program materials, including leader manuals, parent resources, and a how-to on delivery the intervention, are available for a licensing fee so that other healthcare teams can offer the PAC intervention in their local area. Healthcare teams interested in the PAC intervention are encouraged to contact the first author (GDCG) with questions and to discuss opportunities for adaptation and implementation.

## Abbreviations

BMI: body mass index
CBT: cognitive behavioral therapy
FST: family systems theory
IM: Intervention Mapping
PAC: Parents as Agents of Change^©^

## Footnotes

1 For additional information regarding session content, please see Table 1 in Ball et al. [71] or contact the first author (GDCB).
